# Haplotype Association Mapping Identifies a Candidate Gene Region in Mice Infected With *Staphylococcus aureus*

**DOI:** 10.1534/g3.112.002501

**Published:** 2012-06-01

**Authors:** Nicole V. Johnson, Sun Hee Ahn, Hitesh Deshmukh, Mikhail K. Levin, Charlotte L. Nelson, William K. Scott, Andrew Allen, Vance G. Fowler, Lindsay G. Cowell

**Affiliations:** * Department of Biostatistics and Bioinformatics and; † Department of Medicine, Duke University Medical Center, Durham, North Carolina 27710; ‡ Department of Pediatrics, University at Buffalo, Buffalo, New York 14222; § Department of Clinical Sciences, University of Texas Southwestern Medical Center, Dallas, Texas 75390; ** Duke Clinical Research Institute, Duke University Medical Center, Durham, North Carolina 27705; †† Dr. John T. Macdonald Foundation Department of Human Genetics and John P. Hussman Institute for Human Genomics, University of Miami Miller School of Medicine, Miami, Florida 33136

**Keywords:** host genetic susceptibility, infectious disease, kallikrein gene family

## Abstract

Exposure to *Staphylococcus aureus* has a variety of outcomes, from asymptomatic colonization to fatal infection. Strong evidence suggests that host genetics play an important role in susceptibility, but the specific host genetic factors involved are not known. The availability of genome-wide single nucleotide polymorphism (SNP) data for inbred *Mus musculus* strains means that haplotype association mapping can be used to identify candidate susceptibility genes. We applied haplotype association mapping to Perlegen SNP data and kidney bacterial counts from *Staphylococcus aureus*-infected mice from 13 inbred strains and detected an associated block on chromosome 7. Strong experimental evidence supports the result: a separate study demonstrated the presence of a susceptibility locus on chromosome 7 using consomic mice. The associated block contains no genes, but lies within the gene cluster of the 26-member extended kallikrein gene family, whose members have well-recognized roles in the generation of antimicrobial peptides and the regulation of inflammation. Efficient mixed-model association (EMMA) testing of all SNPs with two alleles and located within the gene cluster boundaries finds two significant associations: one of the three polymorphisms defining the associated block and one in the gene closest to the block, Klk1b11. In addition, we find that 7 of the 26 kallikrein genes are differentially expressed between susceptible and resistant mice, including the Klk1b11 gene. These genes represent a promising set of candidate genes influencing susceptibility to *Staphylococcus aureus*.

*Staphylococcus aureus* (*S. aureus*) causes a diverse array of clinical conditions in humans, ranging from asymptomatic colonization to endocarditis and death. The factors influencing infection severity, however, are not fully understood. While it is clear that bacterial (Ferry *et al.* 2005) and environmental factors (Laupland *et al.* 2003) influence the type and severity of *S. aureus* infections, an increasing body of evidence suggests that host genetics also play an important role. For example, higher rates of *S. aureus* infection have been observed among genetically distinct populations, including New Zealand Maori (Hill *et al.* 2001), Canadian Native Americans (Embil *et al.* 1994), and Australian Aboriginals (Tong *et al.* 2009). Familial clusters of recurrent *S. aureus* infections (Noble *et al.* 1967; Zimakoff *et al.* 1988) and rare genetic conditions characterized by susceptibility to *S. aureus* (*e.g.* Job Syndrome, Chediak-Higashi Syndrome) (Faigle *et al.* 1998; Hill *et al.* 2001) have also been described. In addition, several recent studies have demonstrated the importance of host genetics in susceptibility to *S. aureus* using inbred strains of mice. For example, von Kockritz-Blickwede *et al.* (2008) showed that inbred A/J mice are highly susceptible to *S. aureus* and C57BL/6J mice are resistant. Ahn *et al.* (2010) localized this susceptibility to *S. aureus* in A/J mice to chromosomes 7, 8, 11, and 18. These studies not only validate the role of host genetics in disease severity but also confirm the utility of using inbred mouse strains to study *S. aureus* infection.

A major benefit to using inbred mice for the study of host genetic susceptibility to infectious diseases is the availability of highly dense, genome-wide single nucleotide polymorphism (SNP) data, such as is available from the Mouse Genome Informatics (MGI) database (http://www.informatics.jax.org/). The availability of these data has enabled the development of computational methods for the identification of genomic regions containing genetic variants associated with a specific disease phenotype (Grupe *et al.* 2001; Kang *et al.* 2008; Pletcher *et al.* 2004; Tsaih and Korstanje 2009), an approach referred to as haplotype association mapping (HAM). Although experimental validation of HAM-identified genomic regions is necessary, HAM offers significant advantages over the use of conventional quantitative trait locus (QTL) mapping alone in the form of reduced time and financial costs and the identification of smaller genomic regions.

HAM has been used successfully both to identify specific disease-associated genes or SNPs within large QTL found through conventional intercross experiments (Cervino *et al.* 2005; Hillebrandt *et al.* 2005) and to identify novel QTL in genome-wide studies (Bopp *et al.* 2010; Burgess-Herbert *et al.* 2009; Rangnekar *et al.* 2006; Smith *et al.* 2003; Yang *et al.* 2009). Despite these successes, HAM has been criticized for having a high false-positive rate resulting from the recent common ancestry of inbred mouse strains (Kang *et al.* 2008) and for having low power (Cervino *et al.* 2007; Payseur and Place 2007). Some studies have focused on reducing the false-positive rate by developing HAM methods that account for population structure (Kang *et al.* 2008; McClurg *et al.* 2007). Others have focused on characterizing the power of the method and the dependence of power on (i) strain selection, (ii) the frequency and effect size of the disease-causing variant, and (iii) local patterns of linkage disequilibrium (Kirby *et al.* 2010; Payseur and Place 2007). Although these limitations must be taken into consideration when designing HAM studies and interpreting results, HAM has proved useful in identifying novel disease-associated genes (Bopp *et al.* 2010; Cervino *et al.* 2005; Hillebrandt *et al.* 2005; Rangnekar *et al.* 2006; Yang *et al.* 2009).

To study the role of host genetics in susceptibility to *S. aureus* infection, we applied HAM to four phenotypes measured in inbred mice infected with *S. aureus*: survival time, bacterial load in the kidney, bacterial load in the peritoneal fluid, and levels of serum interleukin 6 (IL-6). We present the results of this work here and discuss the implications for host genetic susceptibility to *S. aureus*.

## Materials and Methods

### Survival data

Mice from 13 inbred strains (N = 142) (A/J, BALB/cByJ, 129S1/SvImJ, AKR/J, NZW/LacJ, PWD/PhJ, KK/HlJ, FVB/NJ, BTBR T+ tf/J, DBA/2J, C3H/HeJ, NOD/ShiLtJ, and C57BL/6J) were intraperitoneally injected with 10^7^ colony forming units per gram (CFU/g) of the methicillin-susceptible Sanger 476 strain of *S. aureus* (Table 1). Mice were monitored every eight hours for five days, and survival times were recorded in hours. The median survival time was calculated for each strain and used for HAM. Mice were euthanized using CO_2_ asphyxiation if they appeared moribund. Pain and distress were assessed using a numerical scale for the following characteristics: appearance (0 = normal; 1 = lack of grooming; 2 = rough hair coat; 3 = abnormal posture); behavior (0 = normal; 1 = moving slowly; 2 = moving slowly, irregular ambulation; 3 = immobile). A total score (appearance plus behavior) of three indicated significant pain and distress and culminated in the early euthanasia of the animal. A log-rank test was used to detect statistically significant differences between the survival curves.

**Table 1:** Number of mice injected with *S. aureus* for each of 13 inbred mouse strains

Strain	Survival	Kidney Count	Peritoneal Fluid	Serum IL6
A/J	15	10	12	3
BALB/cByJ	10	4	4	3
129S1/SvImJ	10	8	7	3
AKR/J	14	5	4	3
NZW/LacJ	10	5	4	4
KK/HlJ	10	5	4	3
PWD/PhJ	10	4	4	3
FVB/NJ	8	8	3	3
BTBR t+tf/J	10	5	3	3
DBA/2J	10	5	4	3
C3H/HeJ	15	5	4	3
NOD/ShiLtJ	10	3	3	3
C57BL/6J	10	9	12	9
Total	142	76	68	46

### Bacterial load in the kidney and peritoneal fluid

A total of 76 mice from 13 strains were injected with *S. aureus* as described above (Table 1). Mice were killed 24 hr post infection. Kidneys were collected from all 76 mice; peritoneal fluid was obtained from 68 of the 76 mice. Kidneys and peritoneal fluid were collected and processed as previously described (Deshmukh *et al.* 2009). Briefly, kidneys were collected from killed mice and homogenized in phosphate buffered saline (PBS). Kidney homogenate and peritoneal fluid were diluted serially with PBS 10-fold. The serial dilutions (50 µl) were plated on Tryptic Soy Agar plates, incubated (37°) overnight, and bacterial colonies were counted. For both phenotypes, the average number of CFU per either gram or milliliter was calculated for each strain and used for HAM. Single-factor ANOVA was used to detect statistically significant differences between the strain mean phenotype values.

### Serum levels of IL-6

A total of 46 mice from 13 strains were injected with *S. aureus* as described above (Table 1). Mice were killed at 24 hr post infection. Blood was obtained by intracardiac puncture, serum was separated by centrifugation, and the amount of total protein was measured using the bicinchoninic acid method kit (Pierce). Serum samples were diluted to ensure equal amounts of total protein, and the levels of IL-6 were estimated using an enzyme-linked immunosorbent assay (Due kit, Invitrogen). Single-factor ANOVA was used to detect statistically significant differences between the strain mean phenotype values.

### Trait heritability

Broad-sense heritability (*H^2^*) was estimated for the kidney count, peritoneal fluid count, and serum IL-6 phenotypes using

$$H^{2}=\frac{\sigma_{b}^{2}}{\sigma_{b}^{2}+\sigma_{w}^{2}}$$

where $\sigma_{b}^{2}$ is the between-strain variance and $\sigma_{w}^{2}$ is the within-strain variance (Anholt and Mackay 2009). $\sigma_{b}^{2}$ and $\sigma_{w}^{2}$ were estimated from the between-strain and within-strain mean squared errors obtained by single-factor ANOVA. These heritability estimates likely overestimate the true heritability as two of the strains used, C57BL/6J and A/J, were selected for study as representative of extreme *S. aureus* susceptibility phenotypes (Hegmann and Possidente 1981).

### Haplotype association mapping

**Figure 1:**
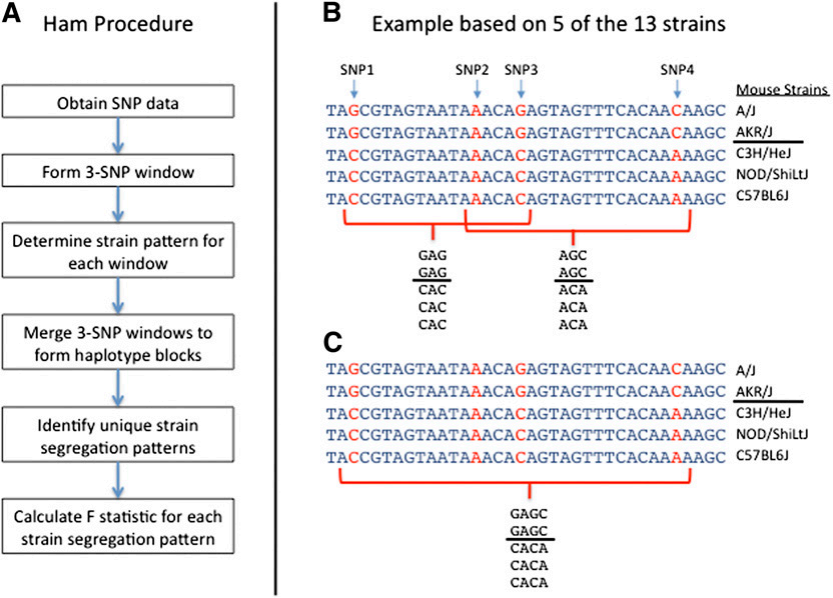
Overview of haplotype association mapping (HAM). (A) Overall HAM approach. (B) Formation of three-SNP windows. (C) Formation of haplotype blocks.

The overall approach is outlined in Figure 1A. Briefly, genotype data were obtained from the Perlegen SNP database (http://phenome.jax.org/db/q?rtn=projects/projdet&reqprojid=198) for the 13 inbred mouse strains for which phenotype data were collected. SNPs with genotype information available for all 13 strains (3,260,963 SNPs) were used for HAM. Overlapping three-SNP windows were formed using three adjacent SNPs, as described in Pletcher *et al.* (2004). For each window, mouse strains were assigned to haplotype groups based on the three SNP alleles within the window, thereby forming the strain segregation pattern for the window (Figure 1B). Adjacent three-SNP windows with the same strain segregation pattern were combined to form a single haplotype block (Figure 1C). Haplotype blocks with only a single haplotype were excluded from the analysis. Among the resulting 1,364,341 haplotype blocks, 66,906 unique strain segregation patterns were observed. For each of the four phenotypes, a test of association was conducted for each pattern using the weighted *F* statistic defined in Pletcher *et al.* (2004).

To determine which strain segregation patterns demonstrate a significant genotype-phenotype association, we applied a modified version of the minP method described in Ge *et al.* (2003). Briefly, for each phenotype, a permutation analysis (described below) was conducted to generate a null distribution of *F* statistics (*F_p_*) for each strain segregation pattern. The *F_p_* distribution for a pattern was used to estimate the type I error (*p_o_*) for the observed *F* statistic (*F_o_*) for that pattern by counting the number of *F_p_* ≥ *F_o_* and dividing by the number of permutations (five million). Type I error (*p_p_*) was estimated for each *F_p_* in the same way. For each permutation, the minimum *p_p_* (*min p_p_*) across all unique strain segregation patterns was captured and used to generate a null distribution of genome-wide minimum *p* values. Genome-wide type I error was then estimated for each phenotype by dividing the total number of *min p_p_* smaller than the minimum *p_o_* (*min p_o_*) for that phenotype by the number of permutations (five million). In addition, for each phenotype, the 5^th^ percentile (*p_pc_*) of the null *min p_p_* distribution was used as the significance threshold for identification of significantly associated genomic regions: haplotype blocks for which the observed *p_o_* was smaller than *p_pc_* were deemed to be significantly associated with phenotype.

A permutation analysis was conducted for each phenotype as follows. For the bacterial count and serum IL-6 phenotypes, a mean and variance were calculated for each strain from the observed phenotype values of the individual mice and were used to parameterize 13 phenotype distributions. For a single permutation, a strain phenotype value was sampled from each of the 13 distributions, and the set of 13 values was permuted. An *F_p_* statistic was then calculated for each of the 66,906 unique strain patterns using the set of permuted strain phenotype values. For the survival phenotype, the median survival times for the 13 strains were permuted. For all four phenotypes, five million permutations were run.

The permutations were run on the high-performance compute cluster Lonestar at the Texas Advanced Computing Center (http://www.tacc.utexas.edu/) at the University of Texas at Austin.

### Efficient Mixed-Model Association

The population structure and genetic relatedness of inbred mouse strains is known to result in inflated false positives in tests of genotype-phenotype association (Kang *et al.* 2008). Efficient mixed-model association (EMMA) conducts tests for association on single SNPs with two alleles, correcting for population structure and genetic relatedness in model organism association mapping (Kang *et al.* 2008). To assess the likelihood that the genomic region identified by the above-described HAM procedure represents population structure artifact, we used EMMA to conduct a test of association for all two-allele SNPs within the region. We used the publicly available R-package implementation of EMMA (available at http://mouse.cs.ucla.edu/emma/). We corrected for multiple testing using a BH-adjusted *P* value computed using the R package mt.rawp2adjp. The BH option utilizes the Benjamini and Hochberg step-up FDR-controlling procedure (Benjamini and Hochberg 1995).

### Gene expression data

Gene expression studies were conducted on mice from 6 of the 13 inbred mouse strains, the 3 resistant strains (C57BL/6J, NOD/LtJ, and C3H/HeJ) and the 3 highly susceptible strains that had high average kidney count values (A/J, AKR/J, and BALB/cByJ). Three mice from each strain were infected with *S. aureus* as described above, and blood was taken by intracardiac puncture 2 hr after infection. Blood was also taken from three uninfected mice of each strain. Blood was stored in RNAlater at −20°. Total RNA was prepared from mouse blood using the Mouse RiboPure Blood RNA isolation kit (Ambion), and globin mRNA was removed using the Globinclear kit (Ambion). All RNA samples passed the quality criteria of the Agilent Bioanalyzer and were used for the analysis. One round of linear amplification was performed for all samples (Ambion MessageAmp Primier). Biotin-labeled cDNA was hybridized to Affymetrix Mouse Genome 430 2.0 Array-Chips for 16 hr at 45° following the manufacturer’s instruction. The arrays were then washed and labeled with streptavidin-phycoerythrin (strep-PE), and the signal was amplified using biotinylated anti-streptavidin followed by another round of staining with strep-PE. Washing and staining were performed on the Affymetrix fluidics station according to recommended protocols. Labeled gene chips were scanned using an Affymetrix Genechip Scanner 7G.

### Microarray data analysis

Preprocessing was conducted using the Robust Multichip Analysis (RMA) (Irizarry *et al.* 2003) implementation in the Bioconductor “affy” package (http://www.bioconductor.org/), with an additional step to account for differences in probe hybridization resulting from SNPs between susceptible and resistant mice. The additional step is referred to as SNP masking (Walter *et al.* 2007) and is applied after background correction and quantile-quantile normalization but prior to the determination of probeset expression values. Genomic locations hybridized by each probe were obtained from the Ensembl database (http://www.ensembl.org/index.html), and these genomic locations were compared to the locations of SNPs for which at least one susceptible and one resistant strain have different alleles. Probes that hybridize to such SNPs within their target transcripts were excluded from the determination of probeset expression values. Probesets with fewer than four remaining probes were excluded from further analysis.

Twenty-five probesets on the Mouse 430 2.0 array were identified as described in *Results* and analyzed using ANOVA to determine whether there were statistically significant differences in the mean expression levels between susceptible and resistant mice. The following generalized linear model was used:

$$Y_{i,j,k,l}=B_{i}^{1}+B_{j}^{2}+T_{k}+S*T_{k,l}$$

where B^1^ corresponds to IVT batch effects, B^2^ to hybridization batch effects, T to infection main effects, and S*T to strain-infection interaction effects. Two factor levels were used for infection state: uninfected and infected. Two factor levels were used for strain: susceptible and resistant. False discovery rate (FDR)-adjusted *P* values were calculated using a false discovery rate of 0.1 (Storey and Tibshirani 2003).

## Results

### Phenotype varies with mouse strain for inbred mice infected with *S. aureus*

**Figure 2:**
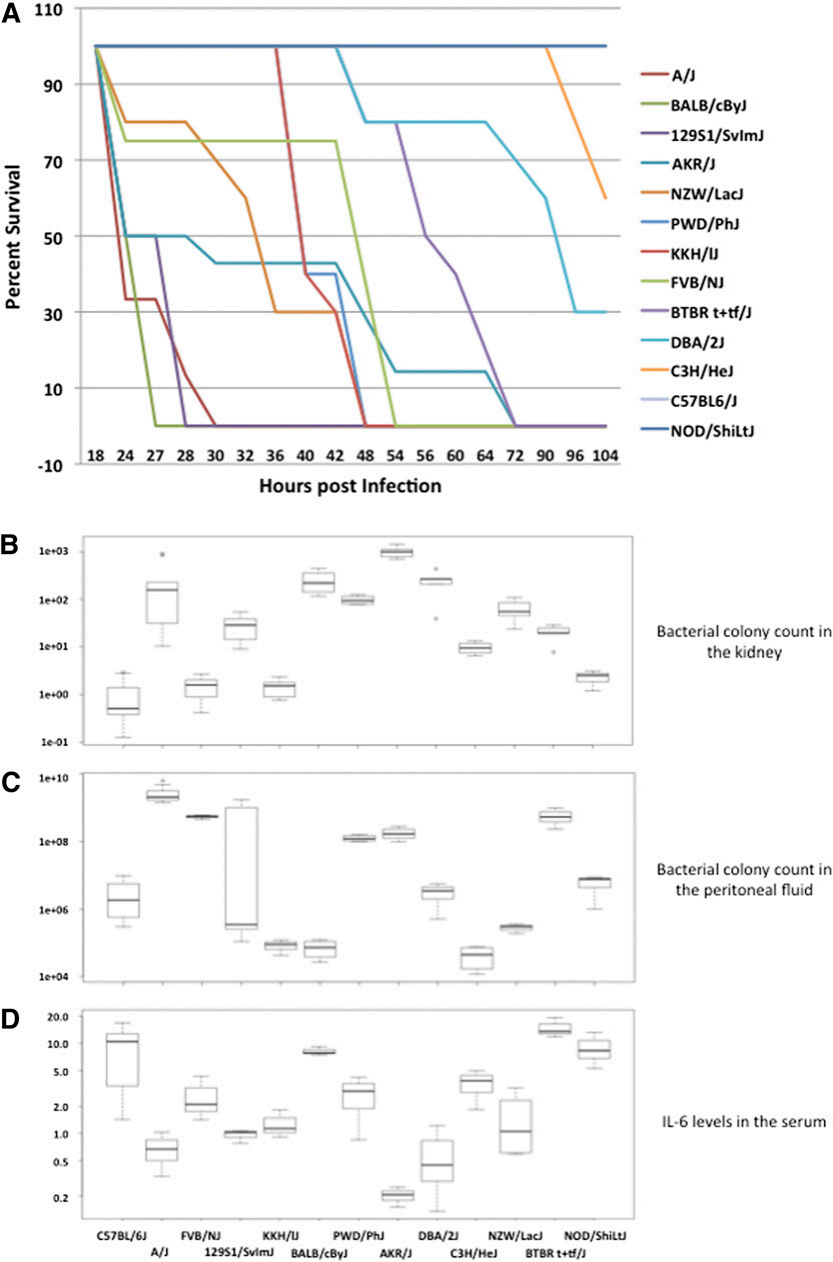
Phenotype data obtained from 13 inbred mouse strains injected with *S. aureus* illustrate a wide spectrum of susceptibility to *S. aureus* infection. (A) Survival curves for each mouse strain. Percentage survival is shown on the y-axis, and survival time, in hours, is shown on the x-axis. The legend lists strains in ascending order of median survival time. (B, C) Box-and-whisker plots of the log_10_ bacterial colony count obtained from the kidney (B) and the peritoneal fluid (C). Colony forming units per gram (CFU/g) or milliliter (CFU/ml) are shown on the y-axis, and strain is shown on the x-axis. (D) Fold change in serum IL-6 levels in mice 2 hr after infection relative to uninfected mice of the same strain. Fold change is shown on the y-axis; other details as described in part B.

A wide range of values was observed for all four phenotypes measured in this study (Figure 2 and File S1). With regard to survival, there were three resistant strains for which none of the mice died (C57BL/6J, NOD/ShiLtJ, and C3H/HeJ), and four highly susceptible strains with median survival times that were ≤ 26 hr (A/J, BALB/cByJ, 129S1/SvMJ, and AKR/J) (Figure 2A). The remaining six strains exhibited intermediate median survival times (Figure 2A). A log-rank test indicated a statistically significant difference between the survival curves (*P* < 10^−16^).

The three resistant strains, along with KK/HlJ and FVB/NJ, had low average kidney count values (<10 CFU/g), whereas three of the highly susceptible strains, along with DBA/2J, had high average kidney count values (≥244 CFU/g) (Figure 2B). The remaining four strains, including the highly susceptible strain 129S1/SvImJ, had average kidney count values ranging from 20 CFU/g to 97 CFU/g (Figure 2B). Single-factor ANOVA indicated statistically significant differences with *F* = 17.03 and *P* = 1.87 × 10^−15^ (numerator and denominator degrees of freedom are 12 and 63, respectively). Broad-sense heritability is estimated to be 0.94.

The highest average peritoneal fluid counts were observed for the A/J, 129S1/SvMJ, AKR/J, PWD/PhJ, FVB/NJ, and BTBR T+ tf/J strains (>1258 CFU/m), whereas the BALBcBy/J, NZW/LacJ, KK/HlJ, and C3H/HeJ strains all had very low average peritoneal fluid counts (<3 CFU/ml) (Figure 2C). The remaining three strains had moderate average peritoneal fluid counts ranging from 33 CFU/ml to 58 CFU/ml (Figure 2C). Single-factor ANOVA indicated statistically significant differences with *F* = 10.52 and *P* = 2.21 × 10^−10^ (numerator and denominator degrees of freedom are 12 and 55, respectively). Broad-sense heritability is estimated to be 0.91.

The NOD/ShiLtJ, BALBcBy/J, C57BL/6J, and BTBR T+ tf/J strains had relatively large average fold increases in serum IL-6 levels (>8), whereas the A/J, 129S1/SvImJ, AKR/J, and DBA/2J strains all had average fold changes that were <1 (Figure 2D). The remaining five strains had moderate fold increases ranging from 1 to 4 (Figure 2D). Single-factor ANOVA indicated a statistically significant difference with *F* = 7.06 and *P* = 3.84 × 10^−6^ (numerator and denominator degrees of freedom are 12 and 33, respectively). Broad-sense heritability is estimated to be 0.88.

When comparing the highly susceptible and resistant strains, we observe that the highly susceptible A/J and AKR/J strains have similar phenotype values: low median survival times, high average bacterial counts in the kidney and peritoneal fluid, and low average levels of serum IL-6. Among the three resistant strains, we find that the NOD/ShiLtJ and C57BL/6J strains have similar patterns: no deaths observed within five days post infection, low average kidney counts, moderate average peritoneal fluid counts, and high average levels of serum IL-6.

### HAM identifies a haplotype block on chromosome 7 significantly associated with kidney bacterial counts

For each phenotype, we identified the smallest HAM *p* value genome-wide, *min p_o_* (Table 2). We found that, for the kidney count phenotype, the *min p_o_* represents a significant association between phenotype and a single haplotype block 1.9 kb in length located on chromosome 7 at 51,256,409–51,258,299 bp (B_7_). This block also has the genome-wide *max F_o_* for the kidney count phenotype. No other block was significantly associated with the kidney count phenotype, and none of the other phenotypes was found to be significantly associated with any block.

**Table 2:** Genome-wide minimum observed *p* value and associated type I error estimate for each phenotype

Phenotype	*min p*_o_	*F_o_*	Type I Error for *min p_o_*
Median survival	0.000042	40.08	0.53
Serum IL6	0.000187	63.51	0.68
Peritoneal fluid	0.000055	99.80	0.24
Kidney	0.000002	948.32	0.02

**Figure 3:**
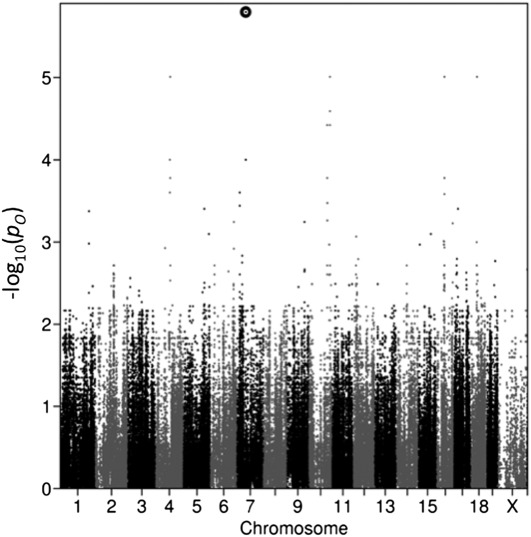
The distribution of –log_10_(*p_o_*) throughout the genome. For each haplotype block, one black dot is plotted for the block’s –log_10_(*p_o_*) value. Location is shown on the x axis; the x-axis coordinate of each dot corresponds to the middle of the block. The black circle at the top indicates the –log_10_(*p_o_*) corresponding to the block B_7_, with a statistically significant association with the kidney count phenotype.

The distribution of *F_o_* and *p_o_* throughout the genome (Figure 3) reveals that the *F_o_* corresponding to B_7_ is much larger than the *F_o_* for any other block. Similarly, the *p_o_* for B_7_ is much smaller than the *p_o_* for any other block.

The strain-haplotype pattern for the single significantly associated block B_7_ consists of five haplotypes with the mouse strain assignments shown in Table 3.

**Table 3:** Strain-haplotype pattern for the single significantly associated block B_7_

Haplotype	Mouse Strains
ACT	AKR/J
GTC	NZW/LacJ
GCC	PWD/PhJ
GTT	A/J, BALB/cByJ, DBA/2J
AAT	129S1/SvImJ, BTBR t+tf/J, C3H/HeJ, C57BL/6J, FVB/NJ, KKHlJ, NOD/ShiLtJ

Interestingly, 9 of the 13 mouse strains, those assigned to haplotypes GTC, GCC, and AAT, have an average kidney count value < 100 CFU/g (Table 3). Of the remaining 4 strains, the A/J, BALB/cByJ, and DBA/2J strains have average kidney counts ranging from 243 CFU/g to 264 CFU/g and share the haplotype GTT. The AKR/J strain does not share its haplotype with any other strain (ACT) and has an average kidney count value an order of magnitude higher than any other strain (1013 CFU/g).

The block B_7_ does not contain any genes, but it lies within the cluster of the 26-member extended kallikrein (Klk) gene family of serine peptidases (Table 4). The gene closest to B_7_ is Klk1b11, located 1.2 kb away.

**Table 4:** Genes located in the extended region on chromosome 7 interrogated for candidate genes

Genomic Location (bp)		Gene Symbol	Gene Name	Probeset	Differentially Expressed
50945788	50950906	Klk14	Kallikrein related-peptidase 14		
50967937	50982128	Klk13	Kallikrein related-peptidase 13		
51024267	51028946	Klk12	Kallikrein related-peptidase 12	Y	
51029987	51034628	Klk11	Kallikrein related-peptidase 11	Y	Downregulated
51036424	51040780	Klk10	Kallikrein related-peptidase 10	Y	
51047261	51052126	Klk9	Kallikrein related-peptidase 9	Y	
51052947	51059192	Klk8	Kallikrein related-peptidase 8	Y	
51066814	51071729	Klk7	Kallikrein related-peptidase 7	Y	
51079869	51087172	Klk6	Kallikrein related-peptidase 6	Y	
51097639	51106551	Klk5	Kallikrein related-peptidase 5	Y	
51136542	51141174	Klk4	Kallikrein related-peptidase 4	Y	Upregulated
51189141	51194960	Klk15	Kallikrein related-peptidase 15		
51206034	51210311	Klk1b8	Kallikrein 1-related peptidase b8	Y	Downregulated
51222138	51226686	Klk1b1	Kallikrein 1-related peptidase b1	Y	Downregulated
51231431	51235746	Klk1b9	Kallikrein 1-related peptidase b9		
51251247	51255245	Klk1b11	Kallikrein 1-related peptidase b11	Y	Downregulated
51268048	51272335	Klk1b26	Kallikrein 1-related peptidase b26	Y	
51307660	51312075	Klk1b27	Kallikrein 1-related peptidase b27	Y	
51357662	51361944	Klk1b21	Kallikrein 1-related peptidase b21	Y	
51368043	51372292	Klk1b22	Kallikrein 1-related peptidase b22		
51392137	51396977	Klk1b16	Kallikrein 1-related peptidase b16	Y	
51443606	51447822	Klk1b24	Kallikrein 1-related peptidase b24	Y	Downregulated
51453561	51457721	Klk1b3	Kallikrein 1-related peptidase b3	Y	
51462805	51467124	Klk1b4	Kallikrein 1-related peptidase b4	Y	
51471844	51476073	Klk1b5	Kallikrein 1-related peptidase b5	Y	
51480807	51484987	Klk1	Kallikrein 1	Y	Downregulated

### EMMA

To assess the likelihood that the genomic region identified by the above-described HAM procedure represents population structure artifact or whether the result may be driven by one or more of the Klk genes, we used EMMA to conduct a test of association between the kidney colonization phenotype and the 486 two-allele SNPs within the Klk gene cluster (Kang *et al.* 2008). Two of the SNPs are significantly associated with phenotype (*P* = 0.000003694 and adjusted *P* = 0.0008977). One of the SNPs lies within the Klk1b11 gene, and the other is one of the three SNPs that define the boundaries of the HAM-detected block B_7_.

This result indicates that there is association between the HAM-detected genomic region and phenotype even when the association test is corrected to account for population structure. This result also indicates that polymorphisms in the Klk1b11 gene may be driving the HAM-detected association.

### Seven of the 26 Klk genes are differentially expressed between susceptible and resistant mice

Of the 26 Klk genes, 21 have probesets on the array, and they correspond to 25 probesets. We identified 7 that were differentially expressed at the 2-hr time point (*P* < 0.05 with a false discovery rate of 0.1) (Table 4). Klk4 was upregulated, and Klk1, Klk11, Klk1b1, Klk1b11, Klk1b8, and Klk1b24 were downregulated in mice from susceptible strains relative to those from resistant strains.

## Discussion

Exposure to *S. aureus* has a wide variety of outcomes, and there is strong evidence that host genetics play an important role (von Kockritz-Blickwede *et al.* 2008). We therefore characterized 13 strains of inbred mice for four measures of infection severity and utilized HAM (Pletcher *et al.* 2004) to identify a single region on chromosome 7 significantly associated with colonization of the kidney by *S. aureus* and containing a family of candidate genes.

Across the 13 mouse strains, we observe a wide variety of phenotype values that are consistent with what is known about *S. aureus* pathogenesis and the host immune response. High bacterial counts in the kidneys are indicative of bacterial dissemination and are correlated with kidney dysfunction in mouse strains susceptible to *S. aureus* infection (Deshmukh *et al.* 2009; von Kockritz-Blickwede *et al.* 2008). High bacterial counts in the peritoneal fluid are indicative of deficient host clearing of bacteria (von Kockritz-Blickwede *et al.* 2008). Similarly, low levels of IL-6 may be indicative of a deficient host immune response, as IL-6 is an important mediator of inflammation and activator of neturophils and has been shown to be required for successful defense against bacterial pathogens, such as *Streptococcus pneumoniae* (van der Poll *et al.* 1997) and *Listeria monocytogenes* (Kopf *et al.* 1994). Taken together, these data indicate that the highly susceptible strains do not effectively clear *S. aureus* from the site of infection, do not mount an effective IL-6–mediated inflammatory response, and are subject to extensive bacterial dissemination.

We applied HAM as described in Pletcher *et al.* (2004) to the four sets of phenotype data and detected one significant association, which was between bacterial colonization of the kidney and a single haplotype block, B_7_, on chromosome 7. There is strong experimental evidence that B_7_ is in fact linked to one or more causal variants: in a separate study conducted by our group, the presence of a causal variant on chromosome 7 was demonstrated using consomic mice created from the highly susceptible A/J strain and the highly resistant C57BL/6J strain. Mice from the consomic mouse strain created by replacing the C57BL/6J chromosome 7 with the A/J chromosome 7 (Nadeau *et al.* 2000) were more susceptible than C57BL/6J to *S. aureus* infection, with a median survival time of 2.5 days (Ahn *et al.* 2010).

B_7_ does not contain any genes but lies within the gene cluster of the 26-member extended kallikrein gene family. The kallikrein proteins (KLK) have well-established roles in the degradation of extracellular matrix (ECM), the generation of antimicrobial peptides, and the regulation of immune responses, particularly inflammation [reviewed in Morizane *et al.* (2010), Sotiropoulou and Pampalakis (2010), and Sotiropoulou *et al.* (2009)]. Degradation of ECM facilitates the infiltration by immune cells of the skin and other tissues. Thus, reduced ECM degradation by KLK enzymes could inhibit infiltration of the site of infection by host immune cells. Similarly, enhanced ECM degradation by KLK enzymes could facilitate *S. aureus* dissemination. The proteolytic activities of KLKs are important for the generation of antimicrobial peptides, particularly cathelicidins and defensins, which directly kill microbes as well as influence innate immune response processes. In this case also, reduced KLK activity would weaken the host immune response to *S. aureus*.

KLKs play an important role in the regulation of inflammation, particularly through activation of the IL-1β precursor and the potent vasoactive peptides bradykinin and kallidin (Moreau *et al.* 2005). Thus, alterations of KLK activity could result in dysregulation of the inflammatory response. We and von Kockritz-Blickwede *et al.* (2008) observed increased kidney infiltration by *S. aureus* in susceptible mice relative to resistant mice. In addition, von Kockritz-Blickwede *et al.* (2008) observed increased lung infiltration by *S. aureus* and erythrocytes, as well as evidence of extensive lung hemorrhage in A/J mice. These observations are all consistent with severe, increased microvascular permeability in susceptible mice in response to *S. aureus* infection. Further, von Kockritz-Blickwede *et al.* (2008) observed increased levels of serum bradykinin in A/J mice as well as decreased activated partial thromboplastin time. Both observations provide evidence that susceptible mice experience increased microvascular permeability as a result of increased activation of the kallikrein-kinin or contact system.

Our gene expression microarray results provide further evidence for the role of these genes in mediating host susceptibility to *S. aureus* infection. Given the large number of Klk genes, however, the varied and wide-ranging functions of their gene products, and the complicated patterns of coregulation via reciprocal- and auto-proteolysis, a series of gene-specific experiments at the nucleic acid and protein levels are required for each of the 26 genes to disentangle their precise role in host susceptibility to *S. aureus*.

Our study has some limitations. First, we only detected a significantly associated haplotype block for one of four phenotypes. This is likely due to the fact that the HAM approach assumes that phenotypic similarities between mouse strains result from shared underlying genetic variants. Our study phenotypes may result from interactions between many different genetic variants, each of which is shared by only a subset of the strains exhibiting similar phenotypes. In addition, the current study may lack sufficient power to detect the corresponding causal variants. The mouse strain panel used in this study is relatively small. Although some studies have used a similar number of strains and detected significant associations using HAM (Yang *et al.* 2009), many other studies have used much larger numbers of strains (Bopp *et al.* 2010; Kirby *et al.* 2010). It is thus likely that with a larger mouse strain panel, additional associations would be detected.

Finally, survival times were recorded for only five days, resulting in an underestimate of the median survival times, particularly for the resistant strains. This results in an underestimate of the F-statistic sum of squares for the within- and between-haplotype group variability. Although underestimates of the within-group variability may result in false-positive associations, underestimates of the between-group variability may result in false-negative associations.

Despite these limitations, we were able to identify a genomic region significantly associated with susceptibility to *S. aureus* infection, for which there is strong supporting experimental evidence and which implicates a large gene family whose members are promising candidate genes for future biological validation. Future studies in the mouse will identify specific members of the family with a role in *S. aureus* pathogenesis in the murine host and will elucidate the specific mechanisms by which the gene products confer susceptibility. These studies in the mouse will be followed by studies to evaluate the role of the corresponding orthologous genes in human susceptibility to *S. aureus*.

## Supplementary Material

Supporting Information
