# Supplementary material for: Haplotype Association Mapping Identifies a Candidate Gene Region in Mice Infected With Staphylococcus aureus
Source: G3 (Bethesda). 2012 Jun 1;2(6):693–700. doi: 10.1534/g3.112.002501 (PMC3362298; doi:10.1534/g3.112.002501)
Supplement: Supporting Information [file supp_2_6_693__index.html]

Supporting Information 

# Haplotype Association Mapping Identifies a Candidate Gene Region in Mice Infected With *Staphylococcus aureus*

## Supporting Information for Johnson *et al*, 2012

**Files in this Data Supplement:**

- File S1 - Supporting data (.zip, 4 KB)
